# Delaying antimicrobials for pediatric bone and joint infections: Balancing clinical risks with diagnostic benefits

**DOI:** 10.3389/fped.2022.975221

**Published:** 2022-10-31

**Authors:** Justin B. Searns

**Affiliations:** Department of Pediatrics, Sections of Hospital Medicine & Infectious Diseases, University of Colorado School of Medicine, Children's Hospital Colorado, Aurora, CO, United States

**Keywords:** pediatric, osteomyelitis, septic arthritis, *Staphylococcus aureus*, diagnostics

## Introduction

Osteoarticular infections (OAI), including osteomyelitis (OM) and septic arthritis (SA), are serious infections in children with the potential for profound morbidity and life-altering disability (1, 2). Identifying the causative pathogen allows for targeted antimicrobial treatment and offers diagnostic clarity by ruling out rheumatologic and oncologic conditions that may mimic bone and joint infections (3–5).

OAI causative pathogens are typically identified *via* bacterial cultures of blood specimens and operatively obtained “source” specimens (6). Although sedation and surgery increase costs and risks, collecting source specimens from infected musculoskeletal foci increases the chance of finding a causative pathogen by an additional 37% over blood cultures alone (5–8). However, once an infection is suspected, there may be significant delays before a surgical procedure can be performed due to the need for diagnostic confirmation and barriers to surgical resource mobilization, particularly for OM (6). Due to potential delays, providers debate whether antimicrobial therapy should be withheld prior to operative procedures to increase the diagnostic yield from musculoskeletal specimens. However, there are clinical costs to allowing a presumed infection to continue unabated. In addition, surgical procedures for children with OAIs may be pursued not solely for diagnostic guidance, but also for therapeutic benefit. Specifically, surgical drainage can allow for improved source control of an infected focus that may profoundly augment the efforts of antimicrobial therapy alone, especially in the case of abscesses, toxin-mediated disease, or extensive pyogenic infections. These therapeutic benefits are particularly relevant in SA where urgent drainage of an infected joint is required to decrease the risks of ongoing synovial damage and long-term sequelae.

A recently published consensus guideline in the United States recommends “an intentional, but cautious, delay in initiating antibiotic therapy” for 48 to 72 h in well-appearing children with OM while facilitating operative biopsy of the infectious focus, though this guideline does not yet comment on antibiotic timing for SA. Contrasting the US guidance, Canadian and European guidelines suggest avoiding surgical intervention for uncomplicated OM unless a subperiosteal abscess is present, but do recommend routine aspiration of infected joints for SA prior to antibiotics if practical (9, 10). However, the recommendation to delay antimicrobials while awaiting diagnostic procedures may not be feasible in all clinical settings for all patients, and a nuanced approach that weighs clinical risks against diagnostic benefits is needed to guide decisions for each OAI episode. This review aims to explore the arguments on both sides of this debate by evaluating the clinical risks and diagnostic benefits for withholding antimicrobial therapy before surgical drainage of source specimens in pediatric OAIs.

### Disease pathophysiology and microbiology

OAIs are most often categorized based on acuity and pathogenesis (6, 11). Classically, an arbitrary cut-off of 2 weeks of preceding symptoms differentiates acute infections from subacute or chronic presentations. In addition, infections may be hematogenous or contiguous in nature. Hematogenous infections arise *via* transient bacteremia that seeds the vascularized growing musculoskeletal system of children (3). Alternatively, contiguous infections may develop from penetrating trauma, post-surgical complications, or indwelling musculoskeletal hardware. These categorizations influence suspected pathogens, rapidity of disease progression, and empiric antimicrobial selection which factor into the calculus of whether to temporarily delay antimicrobial therapy at time of presentation.

The majority of children with OAIs are previously healthy and present with acute hematogenous infections (3). For these patients, there is a predictable cohort of pathogens with a musculoskeletal tropism frequently identified *via* culture. *Staphylococcus aureus*, including methicillin-susceptible and methicillin-resistant *S. aureus* (MSSA/MRSA), is the most common cause of acute hematogenous OAI, accounting for >80% of found pathogens in the majority of studies. However, recent investigations have found that *Kingella kingae* is a frequent cause of OAIs as well and in some populations may be more common than *S. aureus* among young children (5, 8, 11–14). *Streptococcus pyogenes* and *Streptococcus pneumoniae* are also commonly implicated culprits, though they presently make up only a minority of identified pathogens (14–17). Although this narrow list encompasses the vast majority of pathogens, there are many potential, though rare, bacterial and fungal sources for bone and joint infections including endemic fungi, *Borrelia burgdorferi*, enteric gram negatives (e.g., *Salmonella* species), and *Neisseria gonorrhea* among adolescents. Opponents of withholding antimicrobial therapy for OAIs point to the predictability of typical pathogens for these infections while those in favor of waiting for surgical specimen collection argue emerging antimicrobial resistance supports the decision to prioritize diagnostic interventions by cautiously withholding antimicrobials for well-appearing patients. A balanced approach is required to guide this decision.

### Clinical risks to antimicrobial delays

The damage to bone tissue from osteomyelitis is a multifactorial process involving necrosis from vascular compromise, direct cellular injury from bacteria, and bone resorption from bacterial-induced osteoclastogenesis (11). Up to 9% of children with OM develop sequelae including pathologic fractures, limb angulation, and limb length discrepancy (18). Similarly, synovial tissue damage from SA can lead to long-term sequelae such as decreased range of motion and chronic arthritic complaints and is particularly concerning for hip and shoulder joints (3, 19–21). Given the rapidity of possible joint damage and subsequent morbidity, expeditious drainage of infected joints is a crucial component of SA treatment specifically. For all OAIs, prompt clinical attention is necessary to abort ongoing tissue injury from bacterial destruction and host inflammatory response.

However, there is limited *in vivo* understanding for the precise timeline of bacterial replication in pediatric bones and joints. Murine models have used bioluminescence imaging to show *S. aureus* concentration peaks 3 days after direct inoculation into living bones (22). When a foreign implant is used in a similar model, bacterial progression is slower and peaks by day 11 (23). While these models offer insight into bacterial kinetics for OAIs, there are limited studies in children to better understand the histological and microbiological timeline for disease progression and we have poor insight into the clinical risks associated with each hour of antimicrobial delay.

**Figure 1:**
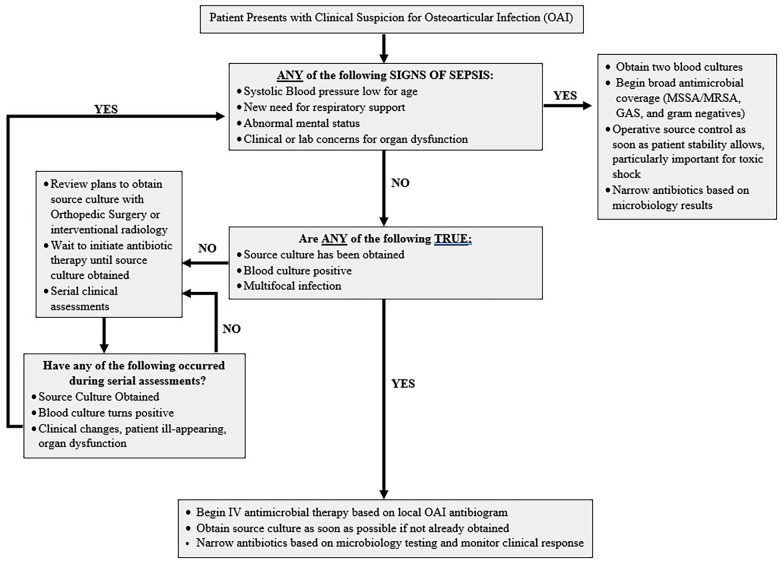
Suggested clinical pathway to follow to help determine need for immediate antimicrobial therapy and strategy to systematically re-evaluate all patients admitted with osteoarticular infections off antimicrobial therapy while expediting surgical intervention.

After our institution's OAI clinical pathway began recommending initiating antimicrobials after surgical drainage for well-appearing patients, there was a delay to first antimicrobial of 6 h (Figure 1). However, despite the brief delay patients had faster fever resolution, faster decline of inflammatory markers, and fewer readmissions (8). This retrospective study suggests there may be minor clinical risks for at least short delays in antimicrobial therapy among well-appearing children with OAIs, though prospective comparisons are lacking for the risks associated with longer delays.

The balance shifts for children with OAIs who present with sepsis, where even brief delays in antimicrobials have severe clinical consequences. Antimicrobial delays of greater than 3 h increase mortality nearly 5-fold among septic children (24). In addition, among critically ill children with OAIs at our institution, we found 100% had a pathogen found *via* blood cultures, decreasing the diagnostic benefit of source cultures in septic patients (25). Therefore, OAI-associated sepsis requires immediate antimicrobial therapy as the consequences of withholding life-saving antimicrobials far outweigh the marginal diagnostic benefit. Instead, source control and operative drainage should be guided by therapeutic benefits to hasten bacterial clearance and prevent toxin-mediated disease. Thankfully, sepsis is rare among pediatric OAIs, and a minority of patients (<5%) present critically ill (18). In addition to sepsis, vulnerable populations (e.g., neonates and immunocompromised children) may have greater clinical risks from delayed antimicrobials. However, these patients may be more likely to be infected with atypical pathogens which increases the benefit from pathogen identification for targeted therapy. Severity of infection and unique patient characteristics must therefore be considered when weighing decisions to withhold immediate antimicrobial therapy for OAIs.

OAIs are a dynamic process, and a patient's clinical symptoms may evolve with time. There is limited guidance on clinical or laboratory markers that can help predict which children with OAIs are likely to decompensate. Therefore, if the decision is made to withhold antimicrobials while surgical resources are mobilized, clinicians should ensure ongoing clinical assessments of the patient to confirm they remain well-appearing. Clinical pathways can promote scheduled reassessments that quickly recognize clinical decompensations and prevent progression to sepsis while awaiting surgery unbeknownst to the treating team (Figure 1) (2, 8, 26, 27).

### Diagnostic benefit to antimicrobial delays

Once infected, the vascularity of musculoskeletal sites decreases which leads to sterilization delays from systemic antimicrobials (7). However, the amount of time before antimicrobials sterilize source cultures remains unknown. Several retrospective single center studies have investigated the impact of antimicrobial pretreatment on bacterial culture yield for bone or joint specimens (5, 7, 12, 28, 29). It seems reasonable that prior antibiotic therapy may decrease the yield of pathogen identification in children with OAIs. For this reason, it is paradoxical that several studies have shown the opposite (5–7, 28, 29). However, most of these studies fail to adjust for confounding, and it is likely that sicker patients with higher bacterial burdens are those most likely to receive immediate antimicrobials. Recently, another retrospective study found antimicrobial pretreatment did not influence culture positivity regardless of duration of antimicrobials after correcting for illness severity (30). However, pretreated patients were still more likely to be febrile and have higher C-reactive protein levels at admission so these findings may similarly be limited by confounding.

There is evidence that the odds of identifying a pathogen *via* source culture decreases with greater antimicrobial exposure. For instance, pretreated patients with positive bone cultures received shorter durations of preceding antibiotics than those with negative cultures (40 h vs. 79 h, *P* = 0.039) (12). Similarly, among 91 children who received antimicrobials prior to musculoskeletal source culture collection, there was a trend towards shorter preceding antimicrobial durations among positive cultures than negative cultures (28.9 h vs. 40.4 h, *P *= 0.1) (5). For children undergoing interventional radiology procedures for bone and joint sampling, there was higher culture positivity among patients who received <24 h of preceding antimicrobials than those who received 24–48 h (90% vs. 50%, *P* = 0.04) (5).

Therefore, it is possible the diagnostic downside of antimicrobial therapy is minimized if we quickly facilitate operative procedures, ideally within 24 h of initiating therapy. In such ideal circumstances, perhaps narrow targeted therapy to treat MSSA, *S. pyogenes*, and *Kingella kingae* (i.e., first-generation cephalosporins), would be reasonable to cover likely pathogens and prevent committing providers to empiric MRSA therapy when cultures remain unrevealing. This strategy might reserve surgical intervention to only those patients who fail to expediently improve with targeted therapy. Such a strategy though may also have unforeseen negative consequences if pretreated source cultures muddy clinical decision-making. For instance, it is plausible when untreated source cultures are negative, providers may pursue diagnostic procedures earlier and more quickly identify atypical pathogens or noninfectious processes in such patients. This concern may be particularly relevant for culture negative SA given the frequent diagnostic challenge of discerning infectious SA from inflammatory or reactive arthritis.

### Considerations for subacute, chronic, or hardware-associated OAIs

For children with subacute, chronic, and contiguous OAIs, the balance of withholding antimicrobials shifts. These patients routinely receive far longer courses of antimicrobial therapy, typically for several months (3, 6). In the case of children with infected musculoskeletal hardware, they may receive a year or longer of suppressive antimicrobial therapy (31). Therefore, the potential benefits of targeted therapy are greater as patients frequently develop adverse events that require adjustments to empiric therapy. In addition, the list of likely causative pathogens for chronic OAIs is more extensive than acute hematogenous infections and includes atypical pathogens. For indwelling bone hardware or contiguous spread from decubitus ulcers, polymicrobial infections become more likely as well. Lastly, by definition these patients present with less rapidly progressive symptoms. Given the greater benefit and likely lower risks, the recommendation to withhold antimicrobials in well-appearing children with non-acute or contiguous OAIs while facilitating surgical intervention is stronger.

### The influence of molecular diagnostics

In the past decade, new diagnostic opportunities have emerged for pediatric OAIs that influence this debate. Several molecular-based microbiologic studies have now been validated for bone and joint specimens in children to target likely causative pathogens. These PCR-based molecular diagnostic tests may be more resilient to the sterilizing effects of antimicrobial pretreatment as they rely on the presence of bacterial genetic material alone instead of a viable pathogen to grow in culture. Furthermore, with the identification of *mecA* as a genotypic marker of methicillin resistance, these PCR-based tests can offer critical antimicrobial resistance information for OAIs specifically.

For bone and joint specimens, the Cepheid^®^ MRSA/SA SSTI PCR was recently found to reliably identify both MSSA and MRSA from musculoskeletal specimens in children (32, 33). In addition, targeted PCRs for *Kingella kingae* are now available for bone and joint specimens (15, 32). A recent proof-of-concept diagnostic study for acute OM in children examined target-enriched multiplex PCR (TEM-PCR) for bone and joint specimens, and found 100% concordance when compared to bacterial culture. In addition, pathogen yield was 80% for TEM-PCR compared to 68% using culture, despite 60% of samples being pretreated with antimicrobials (34). Lastly, new syndromic multiplex PCRs are available that identify a large range of OAI pathogens (35). However, many pathogens included on such panels are more relevant to adult patients (e.g., *Enterococcus faecalis* and *Bacteroides fragilis*).

Finally, non-invasive molecular diagnostic techniques, including microbial cell free DNA next-generation sequencing (mcfDNA-NGS), may offer an opportunity to render this debate obsolete (36). If mcfDNA-NGS of peripheral blood specimens is demonstrated to have a similar yield to invasive surgical source specimens, antimicrobials could be initiated immediately after collection of peripheral blood specimens in children with OAIs. This streamlined ideal process may be within reach, though innovative study designs are needed to determine if non-invasive diagnostic platforms offer promise for OAIs specifically and to investigate where such testing can reliably identify markers of antimicrobial resistance.

### Conclusion and needed future research

OAIs are a heterogeneous group of disorders that require tailored clinical care. For children with OAI sepsis, antimicrobials should be started immediately given the severe clinical risks of delay. For well-appearing children with OM, additional evidence is needed to prospectively compare complication rates and progression to sepsis and to more specifically define what time period is acceptable to withhold antimicrobial therapy while awaiting diagnostic interventions. For well-appearing children with SA, there are clear clinical risks to delaying drainage of an infected joint and therefore surgical procedures for SA should be expedited and highly prioritized from both a diagnostic and therapeutic perspective, which may render the question of antimicrobial delay obsolete in cases of SA under ideal availability of needed surgical resources. Less than 24 h of antimicrobials is less likely to sterilize source cultures, and therefore if antibiotics are initiated, treating teams should quickly expedite surgical procedures to increase the likelihood of microbiological confirmation before bone or joint sterilization. However, there may be undescribed benefits of “truly negative” cultures that are lost when empiric antibiotics are routinely started. Lastly, future studies should determine the impact of antimicrobial therapy on the yield of molecular diagnostic studies of musculoskeletal specimens as these diagnostic tools become more readily available and potentially make this debate obsolete in the near future.

## Author contributions

JBS conceptualized the manuscript, performed the literature review, drafted, revised the final manuscript, contributed to the article, and approved the submitted version.

## References

[1] Gerber JS Coffin SE Smathers SA Zaoutis TE. Trends in the incidence of methicillin-resistant Staphylococcus aureus infection in children's Hospitals in the United States. Clin Infect Dis. (2009) 49(1):65–71. 10.1086/599348 19463065 PMC2897056

[2] Copley LA Kinsler MA Gheen T Shar A Sun D Browne R. The impact of evidence-based clinical practice guidelines applied by a multidisciplinary team for the care of children with osteomyelitis. J Bone Jt Surg. (2013) 95(8):686–93. 10.2106/JBJS.L.00037 23595066

[3] Donaldson N Sanders J Child J Parker S. Acute hematogenous bacterial osteoarticular infections in children. Pediatr Rev. (2020) 41(3):120–36. 10.1542/pir.2018-0201 32123023

[4] Wheeler AM Heizer HR Todd JK. Influence of culture results on management and outcome of pediatric osteomyelitis and/or septic arthritis. J Pediatric Infect Dis Soc. (2012) 1(2):152–6. 10.1093/jpids/pis035 26619168

[5] McNeil JC Forbes AR Vallejo JG Flores AR Hulten KG Mason EO Role of operative or interventional radiology-guided cultures for osteomyelitis. Pediatrics. (2016) 137(5. 10.1542/peds.2015-4616 27244827

[6] Woods CR Bradley JS Chatterjee A Copley LA Robinson J Kronman MP Clinical practice guideline by the pediatric infectious diseases society and the infectious diseases society of America: 2021 guideline on diagnosis and management of acute hematogenous osteomyelitis in pediatrics. J Pediatric Infect Dis Soc. (2021) 10(8):801–44. 10.1093/jpids/piab027 34350458

[7] van der Merwe M Rooks K Crawford H Frampton CMA Boyle MJ. The effect of antibiotic timing on culture yield in paediatric osteoarticular infection. J Chil Orthop. (2019) 13(1):114–9. 10.1302/1863-2548.13.180077 PMC6376441 30838084

[8] Spruiell MD Searns JB Heare TC Roberts JL Wylie E Pyle L Clinical care guideline for improving pediatric acute musculoskeletal infection outcomes. J Pediatric Infect Dis Soc. (2017) 6(3):e86–e93. 10.1093/jpids/pix014 28419275

[9] Saavedra-Lozano J Falup-Pecurariu O Faust SN Girschick H Hartwig N Kaplan S Bone and joint infections. Pediatr Infect Dis J. (2017) 36(8):788–99. 10.1097/INF.0000000000001635 28708801

[10] Le Saux N. Diagnosis and management of acute osteoarticular infections in children. Paediatr Child Health. (2018) 23(5):336–43. 10.1093/pch/pxy049 30653632 PMC6054183

[11] Veis DJ Cassat JE. Infectious osteomyelitis: marrying bone biology and microbiology to shed new light on a persistent clinical challenge. J Bone Miner Res. (2021) 36(4):636–43. 10.1002/jbmr.4279 33740314

[12] Zhorne DJ Altobelli ME Cruz AT. Impact of antibiotic pretreatment on bone biopsy yield for children with acute hematogenous osteomyelitis. Hosp Pediatr. (2015) 5(6):337–41. 10.1542/hpeds.2014-0114 26034165

[13] Chometon S Benito Y Chaker M Boisset S Ploton C Berard J Specific real-time polymerase chain reaction places Kingella kingae as the most common cause of osteoarticular infections in young children. Pediatr Infect Dis J. (2007) 26(5):377–81. 10.1097/01.inf.0000259954.88139.f4 17468645

[14] Verdier I Gayet-Ageron A Ploton C Taylor P Benito Y Freydiere AM Contribution of a broad range polymerase chain reaction to the diagnosis of osteoarticular infections caused by Kingella kingae: description of twenty-four recent pediatric diagnoses. Pediatr Infect Dis J. (2005) 24(8):692–6. 10.1097/01.inf.0000172153.10569.dc 16094222

[15] Ceroni D Cherkaoui A Ferey S Kaelin A Schrenzel J. Kingella kingae osteoarticular infections in young children: clinical features and contribution of a new specific real-time PCR assay to the diagnosis. J Pediatr Orthop. (2010) 30(3):301–4. 10.1097/BPO.0b013e3181d4732f 20357599

[16] Yagupsky P. Kingella kingae: carriage, transmission, and disease. Clin Microbiol Rev. (2015) 28(1):54–79. 10.1128/CMR.00028-14 25567222 PMC4284298

[17] Yagupsky P. Microbiological diagnosis of skeletal system infections in children. Curr Pediatr Rev. (2019) 15(3):154–63. 10.2174/1573396315666190408114653 30961502

[18] Alhinai Z Elahi M Park S Foo B Lee B Chapin K Prediction of adverse outcomes in pediatric acute hematogenous osteomyelitis. Clin Infect Dis. (2020) 71(9):e454–e64. 10.1093/cid/ciaa211 32129457 PMC7904074

[19] Arnold JC Bradley JS. Osteoarticular infections in children. Infect Dis Clin North Am. (2015) 29(3):557–74. 10.1016/j.idc.2015.05.012 26311358

[20] Copley LA. Pediatric musculoskeletal infection: trends and antibiotic recommendations. J Am Acad Orthop Surg. (2009) 17(10):618–26. 10.5435/00124635-200910000-00004 19794219

[21] McNeil JC Vallejo JG Kok EY Sommer LM Hulten KG Kaplan SL. Clinical and microbiologic variables predictive of orthopedic complications following Staphylococcus aureus acute hematogenous osteoarticular infections in children. Clin Infect Dis. (2019) 69(11):1955–61. 10.1093/cid/ciz109 30753346 PMC7348234

[22] Funao H Ishii K Nagai S Sasaki A Hoshikawa T Aizawa M Establishment of a real-time, quantitative, and reproducible mouse model of Staphylococcus osteomyelitis using bioluminescence imaging. Infect Immun. (2012) 80(2):733–41. 10.1128/IAI.06166-11 22104103 PMC3264289

[23] Li D Gromov K Soballe K Puzas JE O'Keefe RJ Awad H Quantitative mouse model of implant-associated osteomyelitis and the kinetics of microbial growth, osteolysis, and humoral immunity. J Orthop Res. (2008) 26(1):96–105. 10.1002/jor.20452 17676625 PMC2701346

[24] Weiss SL Fitzgerald JC Balamuth F Alpern ER Lavelle J Chilutti M Delayed antimicrobial therapy increases mortality and organ dysfunction duration in pediatric sepsis. Crit Care Med. (2014) 42(11):2409–17. 10.1097/CCM.0000000000000509 25148597 PMC4213742

[25] Searns JB DeVine MN MacBrayne CE Williams MC Pearce K Donaldson N Characteristics of children with culture negative acute hematogenous musculoskeletal infections. J Pediatr Orthop. (2022) 42(2):e206–e11. 10.1097/BPO.0000000000002033 34923507

[26] Searns SP, Donaldson N, Sanders J, De S, Soucie C, Stewart J https://www.childrenscolorado.org/49e72a/globalassets/healthcare-professionals/clinical-pathways/musculoskeletal-infection.pdf.

[27] Robinette ED Brower L Schaffzin JK Whitlock P Shah SS Connelly B Use of a clinical care algorithm to improve care for children with hematogenous osteomyelitis. Pediatrics. (2019) 143(1):l. 10.1542/peds.2018-0387 30567715

[28] Ratnayake K Davis AJ Brown L Young TP. Pediatric acute osteomyelitis in the postvaccine, methicillin-resistant Staphylococcus aureus era. Am J Emerg Med. (2015) 33(10):1420–4. 10.1016/j.ajem.2015.07.011 26298052

[29] Section J Gibbons SD Barton T Greenberg DE Jo CH Copley LA. Microbiological culture methods for pediatric musculoskeletal infection: a guideline for optimal use. J Bone Joint Surg Am. (2015) 97(6):441–9. 10.2106/JBJS.N.00477 25788299

[30] Lansell A Vasili Y Suchdev PS Figueroa J Kirpalani A. Impact of antibiotic pretreatment on cultures in children with osteomyelitis and septic arthritis: a retrospective review. BMC Pediatr. (2021) 21(1):342. 10.1186/s12887-021-02806-w 34389010 PMC8361620

[31] Messina AF Berman DM Ghazarian SR Patel R Neustadt J Hahn G The management and outcome of spinal implant-related infections in pediatric patients: a retrospective review. Pediatr Infect Dis J. (2014) 33(7):720–3. 10.1097/INF.0000000000000264 24463805

[32] Searns JB Robinson CC Wei Q Yuan J Hamilton S Pretty K Validation of a novel molecular diagnostic panel for pediatric musculoskeletal infections: integration of the cepheid Xpert MRSA/SA SSTI and laboratory-developed real-time PCR assays for clindamycin resistance genes and Kingella kingae detection. J Microbiol Methods. (2019) 156:60–67. 10.1016/j.mimet.2018.12.004 30527965

[33] Searns JB Parker SK Dominguez SR. Potential Clinical Impact of a Novel Rapid Diagnostic Panel for Pediatric Musculoskeletal Infections Under Review. 10.1093/jpids/piz040 PMC7358044 31194251

[34] Wood JB Sesler C Stalons D Grigorenko E Schoenecker JG Creech CB Performance of TEM-PCR vs culture for bacterial identification in pediatric musculoskeletal infections. Open Forum Infect Dis. (2018) 5(6):ofy119. 10.1093/ofid/ofy119 PMC6007387 29977969

[35] Berneking L Haas M Frielinghaus L Berinson B Lutgehetmann M Christner M Evaluation of a syndromic panel polymerase chain reaction (spPCR) assay for the diagnosis of device-associated bone and joint infections (BJI). Int J Infect Dis. (2022) 116:283–8. 10.1016/j.ijid.2022.01.013 35031396

[36] Blauwkamp TA Thair S Rosen MJ Blair L Lindner MS Vilfan ID Analytical and clinical validation of a microbial cell-free DNA sequencing test for infectious disease. Nat Microbiol. (2019) 4(4):663–74. 10.1038/s41564-018-0349-6 30742071

